# Premature Ventricular Complexes and Left Atrial Appendage Dysfunction - Another Head on a Many-Headed Hydra ?

**DOI:** 10.1016/s0972-6292(16)30646-5

**Published:** 2013-08-01

**Authors:** Raja J Selvaraj

**Affiliations:** Department of Cardiology, Jawaharlal Institute of Postgraduate Medical Education and Research

**Keywords:** Premature Ventricular Complexes, Left Atrial Appendage Dysfunction

Premature ventricular complexes (PVCs) in the absence of structural heart disease are often, but not always benign. They may require treatment for symptoms produced by the PVCs themselves or for intermittent sustained ventricular tachycardia. Even in the absence of symptoms, frequent premature ventricular complexes may lead to the development of left ventricular systolic dysfunction as a form of tachycardia induced cardiomyopathy [1]. Therefore treatment of PVCs is indicated in the presence of LV dilatation and dysfunction or with sufficient frequency of PVCs where development of ventricular dysfunction in the future is considered highly likely. This, however, may not be the only consequence of frequent PVCs.

In an article in this issue of the journal, Patel et al [2] describe a patient with frequent PVCs who developed an embolic stroke. The authors found significantly diminished left atrial appendage (LAA) velocities, especially during frequent ectopy. Left atrial appendage function assessed by flow velocities is well established as a risk factor for stroke in atrial fibrillation, but also to a lesser extent in sinus rhythm [3]. That this diminished left atrial appendage velocity, a marker of left atrial appendage dysfunction, induced by the frequent ventricular ectopy, could have led to thrombus formation with subsequent thromboembolism, is a novel and completely plausible hypothesis, although impossible to verify in this case.

Premature ventricular complexes are associated with loss of atrioventricular synchrony due to their prematurity and inter- and intra- ventricular synchrony due to altered ventricular activation. Thus, they may affect hemodynamics adversely, producing impaired ventricular systolic performance and elevated atrial pressures. Although the risk of thromboembolism with frequent PVCs has not been well studied, it is reasonable to extrapolate from studies that have found higher incidence of atrial fibrillation and thromboembolism in patients with ventricular pacing compared to atrial or dual chamber pacing [4]. It must be noted that the adverse effects of ventricular pacing are linearly related to the percentage of pacing and are seen even with a small percentage of cumulative pacing [5].

One must, however, be cautious about jumping to conclusions from one case. LV dysfunction, by itself, is a determinant of LAA velocity and hence it is difficult to confirm if the diminished LAA velocity was a direct consequence of the frequent PVCs. Premature ventricular complexes are not uncommon in the general population with an estimated prevalence of 1 to 4% [6]. It is unlikely that most of them are at significant risk of thromboembolism from the PVCs. Just as with PVC-induced cardiomyopathy, it is possible that the risk of thromboembolic stroke with PVCs would be a function of the PVC burden [7], the origin of the PVCs [8], possibly the coupling interval and other unknown patient specific factors. More studies are needed to assess the incidence of LAA dysfunction with frequent PVCs and also if this resolves with abolition of the PVCs with ablation or drugs. Risk of stroke associated with frequent PVCs also needs to be studied to evolve guidelines for ablative therapy or anticoagulation in these patients. If there is indeed a subset of patients with frequent PVCs who are at high risk of thromboembolism, this may well turn out to be another reason to get rid of those PVCs even in asymptomatic patients.
